# Validation of the French version of the Revised Diagnostic Interview for Borderlines (DIB-R) for assessing the psychopathology of borderline personality disorder

**DOI:** 10.1186/s40479-023-00233-0

**Published:** 2023-09-18

**Authors:** Ines Culina, Pauline Maillard, Janice Loosli, Chantal Martin-Soelch, Sylvie Berney, Stéphane Kolly, Ueli Kramer

**Affiliations:** 1 Department of Psychiatry, General Psychiatry Service, University Hospital Center and University of Lausanne, Lausanne, Switzerland; 2 Department of Psychology, Unit of Clinical and Health Psychology, University Fribourg, Fribourg, Switzerland; 3 Private Practice, Morges, Switzerland; 4 Institute of Psychotherapy, Lausanne University Hospital, University of Lausanne, Lausanne, Switzerland; 5 Department of Psychology, University of Windsor, Windsor, Canada

**Keywords:** Borderline personality disorder, Assessment, Interview validation, Reliability, Validity

## Abstract

**Background:**

Borderline personality disorder (BPD) is frequently subject to misdiagnosis or underdiagnosis. As a matter of fact, its evaluation poses several challenges, highlighting the importance of having validated evaluation instruments. The Revised Diagnostic Interview for Borderlines (DIB-R) is widely used and recognized for its validity when it comes to assessing the psychopathology of BPD, but, as for now, no French version of the interview exists. The aim of the current work is to validate a French version of the DIB-R.

**Methods:**

The sample consists of *N* = 65 patients with borderline personality disorder (BPD) and *N* = 57 treatment seeking patients (non-BPD comparison group). For inter-rater reliability, a subsample of *N* = 84 interviews will be assessed by two raters, *n* = 47 for the BPD group and *n* = 37 for the non-BPD comparison group.

**Results:**

To assess reliability, we conducted analyses of internal consistency and inter-rater reliability. The results were good for the overall interview as well as for the four domains of the DIB-R. To assess validity, we calculated the receiver operating characteristic (ROC) curve, sensitivity, specificity, predictive values, convergent and discriminative validity. The optimal cutoff was found to be 7. Regarding convergent validity, we found strong convergence between the Borderline Symptom List (BSL-23) and the DIB-R total score. Additionally, the two groups statistically differed on all the DIB-R scores, which indicates that the interview discriminates between the two groups.

**Conclusions:**

Our results indicate good psychometric properties of the French version of the DIB-R. This has important implications as the interview is useful both in clinical settings and for research purposes. Additionally, the present paper aims to contribute to the more general effort of demonstrating generalizability and transportability of the scale.

Borderline personality disorder (BPD) is a highly common mental disorder in the general population and even more so in clinical settings [1–3]. The consequences of BPD can be severe, such as destructive behaviors and suicide attempts, difficulties in relationships, functional impairment and extensive treatment utilization [4, 5]. Correctly identifying BPD is crucial in order to tailor treatment planning [6]; for instance, compared to other mental disorders, pharmacology seems to have little to no success for the improvement of BPD symptoms [7, 8]. Despite its frequency and the serious consequences it can cause, BPD is often underdiagnosed or misdiagnosed [9, 10]. The underlying reasons explaining the difficulties encountered with the BPD diagnosis are various. A first reason can be found in the high comorbidity rates of BPD; the common comorbid disorders are multiple and include mood and anxiety disorders, trauma and stressor-related disorders, substance use disorders as well as other personality disorders [11–14]. When one or more comorbid disorders are present, which is extremely frequent in samples with BPD [15, 16], the clinical presentation is usually complex and it can be hard to disentangle each disorder, especially because symptoms of other disorders can overlap and mask BPD manifestations. Moreover, it is difficult to establish clear boundaries of BPD [17]. For instance, symptoms of bipolar disorder and BPD are commonly confused by clinicians and it is not rare for patients with BPD to receive a wrong diagnosis of bipolar disorder, especially when BPD and major depressive disorder are comorbid [18, 19]. Additionally, BPD is a heterogeneous disorder, to illustrate this statement it is enough to think that there exist 256 possible combinations of criteria if the minimum number of criteria for BPD diagnosis is met, meaning that this number increases if more criteria are present [20]. It is also important to consider that it is common for people to seek treatment during a life crisis or when symptoms of comorbid disorders are particularly severe, making it even more difficult to correctly identify BPD [21]. Lastly, one more factor that could contribute to the underdiagnosis of BPD is the stigma associated to it and thus the hesitation to give this diagnosis [22, 23]. For the above mentioned reasons and because of involuntary possible biases, an evaluation based only on clinical judgment is highly challenging and can be unreliable [21]. In fact, it has been shown that there is a discrepancy between clinical and research practices and that assessment methods impact the frequency of BPD diagnosis [14, 24, 25]. Because of undetected or misdiagnosed BPD, clients often cannot benefit from appropriate psychotherapy and risk being prescribed excessive pharmacological therapy [10, 26].

In light of these elements, it is crucial to reflect on how to facilitate identifying BPD. In order to deal with the diagnostic challenge of this specific personality disorder, a number of semistructured interviews has been developed to be used in research and clinical practice; a systematic review of their validity and reliability has been conducted by Carcone and colleagues [27]. Among them, one commonly used and widely recognized measure is the Revised Diagnostic Interview for Borderlines (DIB-R) [28]. Its first version was developed in the 1970s with the aim of having a measure that would allow to systematically diagnose BPD in a standardized way [29]. The revised version was then published in 1989 with the goal of better discriminating between people with a borderline diagnosis and people with a different personality disorder. One of the particularly relevant and useful aspects of the DIB-R is that it measures a wide variety of clinical manifestations during the two previous years and it offers a detailed insight of the person’s functioning. More specifically, the DIB-R measures four domains of BPD psychopathology, which are affect, cognition, impulse action patterns and interpersonal relationships. The scores of each sub-section are summed up to obtain a total score. Psychometric properties of the original DIB-R are available for the English interview [28, 30], subsequently a Chinese and two Spanish validations have been published [31–33] but, as for now, no French validation of the interview exists.

The aim of the current work is to validate a French version of the DIB-R. Given the challenges surrounding the BPD diagnosis and considered the importance of validated assessment instruments for accurate and prompt identification of BPD, there is the necessity to validate assessment instruments to adapt them to a variety of cultural and linguistic contexts. Additionally, linguistic validations contribute to expand the current evidence on validity and reliability of such measures. With the present work, the hope is to participate to the continuous effort to improve assessment practices and interventions for BPD in French-speaking contexts.

## Methods

### Participants

A total of 122 participants were recruited at a French-speaking University Hospital; more specifically, the sample was composed of two sub-groups: a BPD group with *n* = 65 participants and a non-BPD comparison group with *n* = 57 participants. Mean age for the total sample was 34.4 (*SD* = 12.1) years old, and 78 participants were female (64%). Inclusion criteria were being between 18 and 65 years old, having a sufficient level of French and an indication to have psychotherapy at inclusion time. The BPD diagnosis according to the DSM-5 determined to which group participants would be assigned. Personality disorders were assessed using the structured clinical interview for DSM-5 (SCID-5-CV) [34]; while other comorbid disorders relied on clinicians’ evaluations. Exclusion criteria for all the participants were the presence of a DSM-5 psychotic disorder and mental retardation; exclusion criteria for the comparison group was the presence of a BPD diagnosis. Due to missing data, slightly smaller samples were available for the Borderline Symptom List (BSL-23) [35], the self-reported questionnaire evaluating borderline symptoms: *n* = 50 participants in the BPD group and *n* = 46 in the non-BPD group. Sociodemographic and diagnostic characteristics of the sample are presented in Table 1.

**Table 1:** Sociodemographic and diagnostic characteristics of the BPD and non-BPD groups (*N* = 122)

Variable	BPD group (*n* = 65)	Non-BPD group (*n* = 57)
	Mean or frequency (*SD* or %)	Mean or frequency (*SD* or %)
Age	34.05 (11.45)	34.75 (12.91)
Sex, female	49 (75.4%)	29 (50.9%)
Marital status		
*No partner*	35 (54%)	25 (44%)
*In a relationship /Married*	30 (46%)	32 (56%)
Years of education	13.06 (3.36)	14.79 (3.09)
Currently working or studying	36 (55%)	46 (81%)
Mood disorders	25 (38%)	24 (42%)
Anxiety disorders	12 (18%)	7 (12.3%)
Obsessive–Compulsive and Related Disorders	2 (3%)	2 (3.5%)
Posttraumatic Stress Disorder	1 (1.5%)	1 (1.75%)
Adjustment disorder	1 (1.5%)	13 (22.8%)
Eating disorders	16 (24.6%)	2 (3.5%)
Substance-Related and Addictive Disorders	24 (37%)	2 (3.5%)
Somatic symptoms and related disorders	1 (1.5%)	1 (1.75%)
BPD	65 (100%)	0 (0%)
Paranoid PD	4 (6%)	0 (0%)
Antisocial PD	2 (3%)	0 (0%)
Histrionic PD	0 (0%)	1 (1.75%)
Narcissistic PD	5 (7.7%)	0 (0%)
Avoidant PD	2 (3%)	3 (5%)
Dependent PD	1 (1.5%)	0 (0%)
Unspecified PD	0 (0%)	3 (5%)

*Abbreviations*: *BPD* Borderline personality disorder, *PD* Personality disorder

### Procedure

The research was approved by the local ethics board (number 2016–02235). The validation of the French DIB-R is part of a larger project taking place at a French-speaking Swiss University Hospital that required participants to take part in a research interview during which the DIB-R was administered; participants also had to fill out a series of self-report questionnaires. With a few exceptions, all of the interviews were video-taped, which allowed us to evaluate inter-rater reliability. In fact, in order to evaluate inter-rater reliability, the same interview was assessed by two raters. A total of three raters worked on the project: one PhD student and one research assistant (who was also a licensed psychotherapist) conducted all of the DIB-R interviews; prior to this, they were both trained by the developer and first author of the revised version of the DIB-R. The training consisted of two phases: a first phase where raters assessed interviews carried out by an expert interviewer, and a second phase where raters had to conduct their own interviews with clients. These interviews were video-taped and discussed with trained supervisors. During the whole training process, ratings were discussed with the developer of the scale and all doubts were addressed and resolved. Additionally, a graduate student in clinical psychology received training on how to rate the DIB-R interview with the goal of assessing inter-rater reliability.

### Measures

#### The Revised Diagnostic Interview for Borderlines (DIB-R)

The DIB-R [28] is a semi-structured interview assessing domains relevant to borderline personality disorder, the interview focuses on the two years preceding the interview. It comprises of a total of 127 items, which include summary statements, section scores and two miscellaneous items. The interview groups questions in 22 summary statements, which are in turn part of four global domains: affect (e.g. chronic/major depression; chronic loneliness/emptiness), cognition (e.g. odd thought/unusual perceptual experiences), impulse action patterns (e.g. substance abuse; self-mutilation) and interpersonal relationships (e.g. intolerance of aloneness; recurrent problems in close relationships). Items and summary statements can be rated as 2 (Yes), 1 (Probable) and 0 (No). The scores of the single items will determine the score of the summary statements. The sum of the scores of the summary statements evaluating the same domain represents the domain’s total score, which will then be transformed into a scaled section score following the instructions reported in the interview. The total score is obtained by summing up the four scaled section scores. The total score ranges from 0 to 10 and a score of 8 or more is indicative of the presence of BPD.

Regarding the French version of the DIB-R, the panel of experts involved in the translation process of the interview was composed of 4 psychologists and psychiatrists working both as clinicians and researchers, furthermore, a fifth person participated and assisted in the translation process. Parallel to the translation phase, the four members of the panel followed a 10-session training, each session lasting 1 h, with the first author of the original revised version [28]; this ensured that, by the end of the translation process, every panel member was trained to correctly administer the interview, in addition to already being an expert on the topic of personality disorders. The translation process was as follows: the DIB-R was first translated into French by one of the main researchers working on the project. Subsequently, the translated version was independently reviewed by each panel member and returned to the initial translator; all potential issues regarding wording, cultural appropriateness, clinical accuracy, and comprehension were thoroughly discussed during several meetings until a unanimous consensus on each interview item was reached; an additional fifth person, who was a licensed therapist, contributed to this part of the process. Lastly, the final translation was approved by the first author of the original version of the DIB-R.

#### The Borderline Symptom List (BSL-23)

The BSL-23 [35] is a 23-item self-report questionnaire that evaluates borderline symptoms in adults during the previous week. Items are rated on a Likert-scale ranging from 0 (Not at all) to 4 (Very strong). In order to assess the severity of borderline psychopathology, the overall mean score is calculated. Cronbach’s alpha for the current sample was α = 0.958.

### Statistical analyses

All analyses were carried out using IBM SPSS Statistics, version 27. First, internal consistency of the total score as well as internal consistency of the four subscales of the French DIB-R were calculated using Cronbach’s alphas coefficients. Second, intraclass correlation coefficients were assessed in order to evaluate inter-rater reliability for the total score and the four subscales scores of the French DIB-R. Third, the following indicators were calculated to determine the optimal cutoff point: the receiver operating characteristic (ROC) curve, sensitivity, specificity, positive predictive value (PPV) and negative predictive value (NPV). Fourth, convergent validity was calculated by evaluating the association of the French DIB-R total score with the BSL-23 [35] using Spearman’s correlation. Fifth, we assessed discriminative validity in order to evaluate the French DIB-R ability to discriminate between people who have received the BPD diagnosis and clients who are in psychotherapy and do not have a BPD diagnosis. To do so, we ran independent t-tests between the two groups.

## Results

### Internal consistency

In order to assess internal consistency, Cronbach’s alphas were calculated for the overall interview and for the four domains evaluated by the DIB-R (affect domain, cognition domain, impulse action patterns domain and interpersonal relationships domain). As depicted in Table 2, the results were good, with Cronbach’s alphas higher than 0.84 for all dimensions and for the overall interview.

**Table 2:** Reliability of the French DIB-R

	Internal consistency		Inter-rater reliability					
	Full sample (*N* = 122)		Full sample (*N* = 84)		BPD group (*n* = 47)		Non-BPD group (*n* = 37)	
	N. of items	Cronbach’s alpha	ICC(2; 1)	95% CI	ICC(2; 1)	95% CI	ICC(2; 1)	95% CI
Affect	23	.892	0.97	0.94–0.98	0.98	0.97–0.99	0.96	0.92–0.98
Cognition	30	.880	0.93	0.87–0.96	0.96	0.93–0.98	0.88	0.79–0.93
Impulse action patterns	22	.842	0.94	0.89–0.97	0.97	0.93–0.98	0.90	0.81–0.95
Interpersonal relationships	41	.911	0.93	0.89–0.96	0.96	0.93–0.98	0.89	0.84–0.95
Overall interview	116	.957	0.96	0.94–0.97	0.98	0.96–0.98	0.94	0.91–0.96

ICC estimates and their 95% confident intervals were calculated based on a single-rating, absolute-agreement, 2-way random-effects model interval for a randomly chosen subsample of *n* = 84 participants (69% of the total sample, *N* = 122)

### Inter-rater reliability

A total of 84 participants (69% of the total sample) was assessed by two raters, more precisely, 37 participants of the non-BPD group and 47 participants of the BPD group. In order to assess inter-rater reliability, intraclass correlation coefficients were estimated based on a single-rating, absolute-agreement, 2-way random-effects model. ICC were calculated for the total DIB-R score as well as for the four domains (affect, cognition, impulse action patterns and interpersonal relationships). As presented in Table 2, the inter-rater reliability can be interpreted as being excellent [36].

### Sensitivity and specificity

**Fig. 1:**
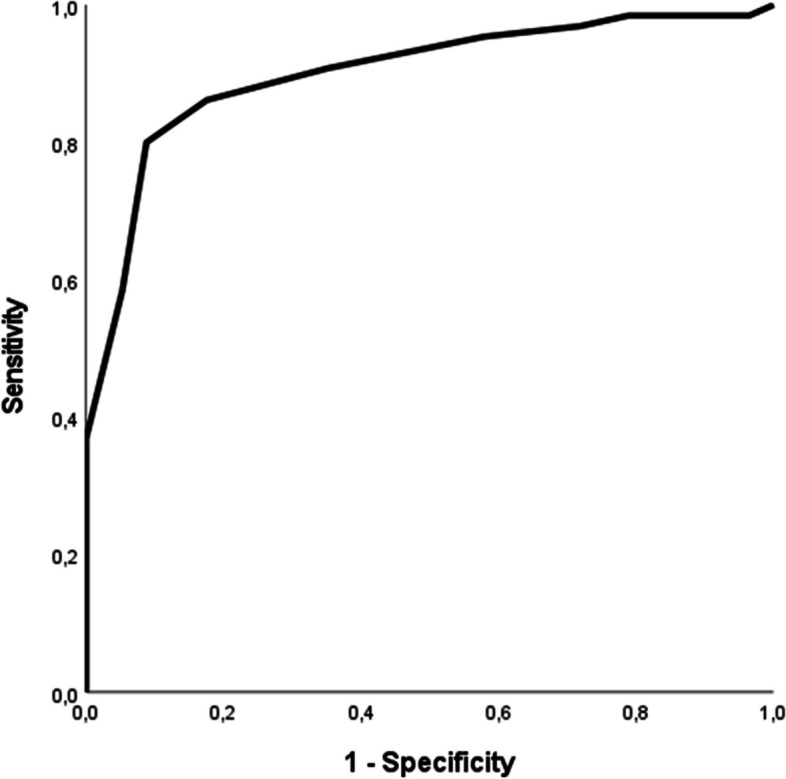
ROC curve of the French DIB-R Note: The area under the curve (AUC) was of .901, 95% CI [.845, .957], *p* < 001

In order to determine the optimal cutoff point allowing to discriminate between people with a BPD diagnosis and people without BPD, we evaluated the ROC curve, which is depicted in Fig. 1. The area under the curve (AUC) was of 0.901, 95% CI [0.845, 0.957], *p* < 001. Additionally, the following indicators were evaluated to establish the best cutoff for the interview: sensitivity, specificity, PPV and NPV. As shown in Table 3, three potential cutoffs showed adequate results and were taken into consideration. The original version of the DIB-R has a cutoff of 8 [28], however, in the current research, this cutoff showed the least satisfactory results among the three, therefore it was rejected. Even though a cutoff of 6 yielded convincing results, we consider that a cutoff of 7 is the most suitable choice for the French version of the interview as it follows a balanced approach between research rigor and clinical relevance. At a cutoff of 7, the interview has a sensitivity of 0.800, 95% CI [0.724, 0.864], a specificity of 0.912, 95% CI [0.861, 0.944], a PPV of 0.912, 95% CI [0.850, 0.950] and a NPV of 0.800, 95% CI [0.734, 0.856]. In fact, these results are robust, furthermore, a cutoff of 7 aligns with the previous linguistic validations [31, 32] and maintains some consistency with the original version [28].

**Table 3:** Sensitivity, specificity, positive predictive value (PPV) and negative predictive value (NPV) of three potential cutoffs of the French version of the DIB-R interview

Cutoff value of the interview	Sensitivity (95% CI)	Specificity (95% CI)	PPV (95% CI)	NPV (95% CI)
≥ 6	.862 (.796 – .913)	.825 (.756 –.881)	.848 (.775 – .902)	.839 (.764–.903)
≥ 7	.800 (.724 – .864)	.912 (.861 – .944)	.912 (.850 – .950)	.800 (.734 – .856)
≥ 8	.692 (.604 – .765)	.930 (.883 –.970)	.918 (.865 – .953)	.726 (.661 – .783)

*CI* Confidence interval, *PPV* Positive predictive value, *NPV* Negative predictive value. According to clinical and statistical criteria, a cutoff of 7 was selected for the French DIB-R

### Convergent validity

In order to evaluate convergent validity we performed a correlation test. More specifically, considering that data was not normally distributed, we performed a non-parametric correlation. The BSL-23 total score and the French DIB-R total score were found to be significantly correlated, *rs*(94) = 0.60*, p* < 0.001. As expected, the higher the score of the BSL-23 (i.e. self-reported questionnaire measuring borderline symptoms), the higher the total score of the DIB-R.

### Discriminative validity

In order to compare the DIB-R scores of the BPD group and the non-BPD group, independent t-tests were performed. The following scores of the DIB-R were compared: affect domain score, cognition score, impulse action patterns score, interpersonal relationships score and the total DIB-R score. As presented in Table 4, all comparisons showed significant results (*p* < 0.001), with Cohen’s d indicating effect sizes ranging from 1.86 to 3.08.

**Table 4:** Differences between the BPD group and NON-BPD group on the French DIB-R (*N* = 122)

	BPD		Non-BPD					
Domain	M	SD	M	SD	*df*	*t*	*p*	Cohen’s d
Total score	8.11	2.31	3.98	2.33	120	-10.59	< 001	2.25
Affect	8.97	2.05	7.40	2.55	120	-3.71	< 001	2.29
Cognition	4.92	1.72	2.67	2.01	120	-6.62	< 001	1.86
Impulse action patterns	5.46	2.44	2.21	1.87	120	-8.32	< 001	2.19
Interpersonal relationships	9.88	3.47	4.65	2.57	120	-9.35	< 001	3.08

*M* Mean and *SD* Standard Deviation. For each domain, average scores were computed. The total score of the DIB-R, the affect domain score and the impulse action pattern domain score range from 0 to 10, the cognition domain score ranges from 0 to 6, the interpersonal relationships domain score ranges from 0 to 18. Non-BPD participants are outpatients at a French-speaking university clinic who do not have borderline personality disorder

## Discussion

The aim of the present study was to validate a French version of the DIB-R, as no French translation of the interview has been validated so far. In fact, to our knowledge, the only validated translations of the DIB-R that exist to date are in Chinese [31] and in Spanish [32, 33]. As stated in the introduction, it is relevant to validate the DIB-R in other languages as it is a detailed instrument which is commonly used and can prove to be useful both in research and in clinical practice. Furthermore, such an instrument can play a particularly crucial role in the screening and diagnostic phase of BPD. As a matter of fact, one of the first challenges encountered with BPD is correctly diagnosing it; misdiagnosis are frequent and can negatively impact treatment planning. Furthermore, BPD is frequent and can lead to serious consequences, which justifies even more the necessity of having validated measures in different cultural contexts.

Overall, the results of the present article are in line with the results of other studies on the validation of the DIB-R [28, 30–33]: the psychometric properties are more than satisfactory and confirm that the French version is adequate and can be used to assess BPD.

Reliability indices are good, more precisely, we assessed internal consistency and inter-rater reliability for the whole interview as well as for the four specific domains assessed by the DIB-R: affects, cognition, impulsive action patterns and interpersonal relationships. Internal consistency results for the whole interview and the four domains were high; the same goes for the agreement between evaluations done by different raters as inter-rater reliability indices were in the excellent range for the overall interview and for the four domains. The reliability results of our study are high and can be considered equivalent to the ones found by the developers of the scale [30]. This is of particular interest as, in their article [30], Zanarini and her colleagues raise the question about the possibility that their reliability indices might not be generalizable to other studies, the reason being that developers of scales assessing personality disorders often obtain more satisfactory results in regards to reliability than other research groups [37]. The results of the current study seem to prove that with the right amount of training and supervision, results can be more than good and even comparable to the ones found by the original research team.

As for validity measures, we assessed the ROC curve, sensitivity, specificity, PPV, NPV, convergent validity and discriminative validity. According to the aforementioned indices used to establish the optimal cutoff for the interview, a cutoff of 7 appeared as the best solution. Statistically, both a cutoff of 6 and a cutoff of 7 would represent an adequate choice. However, several reasons justified our choice. The chosen cutoff aligns with the results of the previous linguistic validations [31, 32] and maintains a degree of consistency with the original version of the interview as it does not diverge excessively from the cutoff of 8 [28]. Furthermore, from a clinical perspective, a cutoff of 7 appears to be more suitable as it has a higher specificity while maintaining a good sensitivity, allowing to detect with efficacy BPD preventing the risk of over diagnosing it, which would possibly be the case with a lower cutoff. In sum, when combining statistical rigor and clinical relevance, a cutoff of 7 seems more suitable for the purposes of the interview. Next, in order to assess convergent validity, we performed correlation analyses between the BSL-23 [35], a self-report measure evaluating the severity of borderline psychopathology. We found a statistically significant correlation that can be interpreted as good. For neither the Spanish [32] nor the Chinese version [31] of the DIB-R convergent validity was calculated, therefore we are not able to compare our results to those of other language validation studies. Zanarini and colleagues examined the convergent validity of the DIB-R by evaluating its relationship with the Zanarini Rating Scale for BPD: their results were satisfactory [38]. Lastly, in order to assess discriminative validity, we compared the scores obtained by the BPD group and the non-BPD group on the overall score and on the four domains of the DIB-R; as described in the results section, a statistically significant difference between the two groups was found for all the aforementioned comparisons with large effect sizes. This seems to indicate that the French DIB-R can be used to successfully discriminate between individuals diagnosed with BPD and a clinical population; these results are in line with those obtained in the Chinese validation study of the DIB-R [31]. Previous research also proved the validity of the DIB-R in discriminating between BPD patients and patients with other personality disorders [39].

Despite the convincing results, the current study has some clear limitations that need to be acknowledged. First of all, the sample size could be too small. Still, we believe that the number of participants was sufficient to conduct a validation study as the sample is comparable to the general number of participants of DIB-R validation studies. Furthermore, there were missing data for the self-report questionnaire (i.e. the BSL-23), even though we had 79% of the sample’s answers, which was deemed a good return rate. Additionally, because of feasibility reasons, we were not able to include test–retest reliability nor longitudinal reliability in our study. Another point that should be raised regards the translation process. In fact, according to a number of guidelines for linguistic validations, back translation is recommended to ensure accuracy of the translated measure [40]. Still, we presume that the quality of our translation is adequate and sufficient for the assessment of BPD in clinical practice as well as in research in French-speaking contexts. As described in the methods section, the translation process was meticulous as the following steps were undertaken: throughout the entire process, the group remained in contact and received training from the first author of the original interview, each member of the panel reviewed the translation independently, final agreement on all items was a requirement for the last version of the translation, which, in addition, had to be approved by the first author of the DIB-R. Lastly, comorbidities were assessed based on clinicians' evaluations and were not measured using validated instruments.

## Conclusions

In conclusion, working with validated measures assessing BPD is important both for research purposes as well as for clinical practice across the globe. With our study, we were able to obtain good psychometric properties for the French version of the DIB-R, which we can recommend to be used to systematically assess psychopathology of BPD in these cultural contexts. Lastly, the present paper contributes to showing the generalizability of the scale across a different linguistic and cultural context.

## Data Availability

The data that support the findings of this study are available from the corresponding author upon reasonable request.
